# *Gambiense* Human African Trypanosomiasis and Immunological Memory: Effect on Phenotypic Lymphocyte Profiles and Humoral Immunity

**DOI:** 10.1371/journal.ppat.1003947

**Published:** 2014-03-06

**Authors:** Veerle Lejon, Dieudonné Mumba Ngoyi, Luc Kestens, Luc Boel, Barbara Barbé, Victor Kande Betu, Johan van Griensven, Emmanuel Bottieau, Jean-Jacques Muyembe Tamfum, Jan Jacobs, Philippe Büscher

**Affiliations:** 1 Institute of Tropical Medicine, Antwerp, Belgium; 2 Institut de Recherche pour le Développement, UMR 177 IRD-CIRAD INTERTRYP, Montpellier, France; 3 Institut National de Recherche Biomédicale, Kinshasa, Democratic Republic of the Congo; 4 Programme Nationale de Lutte contre la Trypanosomiase Humaine Africaine, Kinshasa, Democratic Republic of the Congo; University of Virginia Health System, United States of America

## Abstract

In mice, experimental infection with *Trypanosoma brucei* causes decreased bone marrow B-cell development, abolished splenic B-cell maturation and loss of antibody mediated protection including vaccine induced memory responses. Nothing is known about this phenomenon in human African trypanosomiasis (HAT), but if occurring, it would imply the need of revaccination of HAT patients after therapy and abolish hope for a HAT vaccine. The effect of *gambiense* HAT on peripheral blood memory T- and B-cells and on innate and vaccine induced antibody levels was examined. The percentage of memory B- and T-cells was quantified in peripheral blood, prospectively collected in DR Congo from 117 *Trypanosoma brucei gambiense* infected HAT patients before and six months after treatment and 117 controls at the same time points. Antibodies against carbohydrate antigens on red blood cells and against measles were quantified. Before treatment, significantly higher percentages of memory B-cells, mainly T-independent memory B-cells, were observed in HAT patients compared to controls (CD20+CD27+IgM+, 13.0% versus 2.0%, *p*<0.001). The percentage of memory T-cells, mainly early effector/memory T-cells, was higher in HAT (CD3+CD45RO+CD27+, 19.4% versus 16.7%, *p* = 0.003). After treatment, the percentage of memory T-cells normalized, the percentage of memory B-cells did not. The median anti-red blood cell carbohydrate IgM level was one titer lower in HAT patients than in controls (*p*<0.004), and partially normalized after treatment. Anti-measles antibody concentrations were lower in HAT patients than in controls (medians of 1500 versus 2250 mIU/ml, *p* = 0.02), and remained so after treatment, but were above the cut-off level assumed to provide protection in 94.8% of HAT patients, before and after treatment (versus 98.3% of controls, *p* = 0.3). Although functionality of the B-cells was not verified, the results suggest that immunity was conserved in *T.b. gambiense* infected HAT patients and that B-cell dysfunction might not be that severe as in mouse models.

## Author Summary

African trypanosomes are parasites that cause sleeping sickness in humans. In mice models, trypanosomiasis causes loss of the spleen memory B-cell precursors, of the host memory response and of protection against certain pathogens, built up by vaccination. The phenomenon has never been studied in human sleeping sickness, but if occurring, revaccination after treatment would be required. We show that *gambiense* human sleeping sickness is associated with a relevant increase in memory T- and B- cells in peripheral blood, in particular T-independent memory B-cells. As measles vaccination is included in standard vaccination programs, we measured measles antibody concentrations, which, although slightly lower in sleeping sickness patients than in controls, exceeded in 95% of patients the minimum level considered protective. Anti-red blood cell IgM titres, measured to assess the T-cell independent antibody response, were one titre lower in patients than in controls, but normalized after treatment. Overall, our results in *gambiense* HAT patients do not suggest trypanosomiasis associated massive memory cell destruction, or loss of antibody levels, although the antibody's protective capacity remains to be confirmed.

## Introduction

Human African Trypanosomiasis (HAT) or sleeping sickness, is a vector-borne parasitic disease occurring in sub-Saharan Africa. About 70 million persons are at risk for infection and 30 000 persons are estimated to be infected [1]. The parasites concerned belong to the *Trypanosoma* genus and are transmitted through the bites of tsetse flies (*Glossina* genus). Two subspecies of *Trypanosoma brucei* (*T.b.*), *T.b. gambiense* and *T.b. rhodesiense*, are responsible for human infection, which is usually fatal if left untreated. Infection with *T.b. gambiense* is responsible for chronic HAT in West- and Central-Africa, and characterized by low parasite numbers. In East-Africa, infection with *T.b. rhodesiense* leads to acute disease with relatively high parasite loads. Control of HAT relies on a combination of accurate diagnosis of cases, treatment of detected cases, and on control of the tsetse fly vector. No vaccine is available yet.

The immunopathology of HAT remains poorly understood and most of our understanding comes from experimental *T.b. brucei* infections in mice, which also serve as a model for vaccine development. In *T.b. brucei* infected mice, host control over disease mainly relies on the T-cell independent IgM antibody response [2]–[4]. However, mice *T.b. brucei* infection results in decreased B-cell development in the bone marrow [5]. Lymphopoiesis, which is taken over by the spleen, is in turn abrogated by apoptosis of transitional B-cells, permanent loss of splenic marginal zone B-cells (which are important for the early antibody response against T-cell independent antigens) and depletion of follicular B-cells (which normally develop into antibody producing plasma cells and memory B-cells). As a result of B-cell dysfunction, mice become susceptible to repetitive infections by previously encountered *T.b. brucei* variant antigenic types [6]. Furthermore, *T.b. brucei* infection equally affects the protective immune response towards unrelated pathogens, as observed in two experiments. First, in mice immunized against *Trichinella spiralis*, it was observed that upon subsequent infection with *T.b. brucei* and *Trichinella spiralis*, the effect of vaccination was lost in *T.b. brucei* infected mice only [7]. Similarly, in mice vaccinated against diphtheria, tetanus and *Bordetella pertussis*, the vaccine mediated protective effect was abrogated in mice that were infected with *T.b. brucei* prior to a *Bordetella pertussis* challenge, while vaccinated mice that had not been infected with *T.b. brucei*, remained protected upon challenge with *Bordetella* [6]. *In vivo* and *in vitro* correlates of cell-mediated immunity were observed to be depressed as well in rabbits infected with the African trypanosome *T. congolense* [8].

These results indicate that *T.b. brucei* infections can give rise to general memory B-cell destruction in animals, and point to the possibility that *T. brucei* infection may destruct memory B-cell and abrogate vaccine induced protection in humans as well. If confirmed, this would imply the need of revaccination of HAT patients after anti-trypanosomal therapy and development of a vaccine against the disease might be hard to achieve [9]. However, the relevance of the experimental models for humans remained unknown. Data about leukocyte phenotypes in HAT have remained limited to one study showing increased percentages of CD19+ B-cells and activated B-cells in blood of *gambiense* HAT patients, as well as a relative decrease in memory and effector CD8 T-cells [10]. Evidence for an increased occurrence of vaccine preventable diseases in cured HAT patients is missing, although such relationships may be easily overlooked due to weak surveillance systems in HAT endemic countries. The vaccine-induced memory response in HAT is difficult to assess. Firstly, one is limited to vaccines that provide life-long protection and have been administered to the majority of the population and prior to trypanosomiasis infection. Secondly, loss of protection cannot be tested by challenge with the pathogen. Moreover, HAT mainly occurs in remote rural settings where no standard laboratory infrastructure or electricity is available. Although in *T.b. brucei* animal models, immune depression may occur despite intact antibody levels [7], we selected antibody quantification as an initial, though suboptimal, approach to assess immunological memory, taking into account that so far, nothing is known for the human situation. We opted for iso-agglutinins, which are innate antibodies against A and B carbohydrate antigens on red blood cells [11], as well as for measles vaccine antibodies, as this vaccine is part of the standard vaccination programs [12].

We addressed the following questions: (i) does *gambiense* HAT eliminate peripheral blood memory B-cells; (ii) are peripheral blood memory T-cells affected in *gambiense* HAT (iii) does *gambiense* HAT influence iso-agglutinin levels and antibody levels against measles, and; (iv) are these effects reversible upon cure from *gambiense* HAT?

## Materials and Methods

### Ethics statement

Before enrolment into the study, written informed consent was obtained from adult participants. In the case of minors, an assent was asked for and parents/guardians provided written informed consent. Ethical clearance for the study was obtained from the institutional review board of ITM and the ethical committees of the University Hospital in Antwerp, Belgium (study registration number B30020108363) and of the Ministry of Health of the Democratic Republic of the Congo (DR Congo).

### Study population and specimen collection

*Trypanosma brucei gambiense infected* HAT patients and non-HAT endemic controls were prospectively enrolled (T = 0 months) in the study in DR Congo, Bandundu Province between July and December 2010. Participants were identified during HAT screening activities of the dedicated HAT mobile team of Masi-Manimba, or included at the HAT treatment centres of Masi-Manimba and Bonga-Yasa. Inclusion criteria for HAT patients were the presence of trypanosomes in blood, lymph and/or cerebrospinal fluid (irrespective of disease stage), and being 12 years or older. Exclusion criteria were pregnancy, being previously treated for HAT and being moribund. For each HAT patient, a control was included, fulfilling the following criteria: same gender and age and being and being resident in the same or a neighbouring village. Inclusion criteria for controls were absence of clinical evidence for HAT (no swollen lymph nodes or neurological symptoms), absence of trypanosome specific antibodies in whole blood detected by card agglutination test for trypanosomiasis (CATT) [13]; no trypanosomes in blood detected by the mini anion exchange centrifugation technique [14] and being 12 years or older. Exclusion criteria were identical as for HAT patients.

At enrolment, a crude assessment of the general condition (normal, good, bad, pre-moribund or moribund) was made, based on the participant's ability to eat, walk and take care of himself independently. Participants were questioned for their vaccination history (measles, diphtheria-tetanus-whooping cough, polio, Bacillus Calmette-Guérin (BCG)) and presence of a BCG scar was verified.

Whole blood was collected by venipuncture and collected in 5 ml Cyto-Chex BCT blood collection tubes (Streck, Omaha, NE, USA) and shipped within one week to the Institute of Tropical Medicine (ITM) for phenotyping. From blood sampled on heparin, plasma was prepared that was snap frozen in liquid nitrogen and shipped to ITM where specimens were stored at −70°C until use. Blood taken on EDTA was preserved in an equal volume of GE buffer (6 M guanidine hydrochloride, 0.2 M EDTA, pH 8.0) at ambient temperature until DNA extraction. Thick and thin blood films were prepared and Giemsa stained for malaria diagnosis.

The participants ABO blood group was determined using Eldoncard 2511 (Eldon Biologicals, Gentofte, Denmark). The HIV status was determined using HIV 1/2 STAT-PAK Assay (Chembio, Medford, NY, USA) which, if positive, was followed by Uni-Gold HIV (Trinity Biotech, Wicklow, Ireland), and if positive, by HIV 1/2 Oraquick ADVANCE (Orasure Technologies, Bethlehem, PA, USA) [15]. In participants positive for all 3 serological tests, HIV infection was confirmed *a posteriori* using PCR, following a nested method in an algorithm of three different primer sets in *pol*, *env* and LTR region [16]. CATT was performed on whole blood taken on heparin, and if positive, the plasma end-titre was determined.

HAT was treated following the guidelines of the National HAT Control Program in DR Congo.

HAT patients were revisited six months after treatment, controls at the corresponding time point (T = 7 months). The participant's general condition was re-assessed. Blood taken on heparin and on Cyto-Chex BCT blood collection tubes was processed as described above. All participants were examined for absence of trypanosomes using the mini anion exchange centrifugation technique, and in controls, CATT was repeated.

### Flow cytometry

Whole blood, collected in Cyto-Chex BCT blood collection tubes (Streck, Omaha, NE, USA), was used to study T and B cell subsets by flow cytometry.

B-cells subsets were analysed using mouse anti-human monoclonal antibodies anti-CD45 PerCP (leucocytes), anti-CD20-FITC (B cells), anti-human CD27-APC (IgG1) and anti-human IgM-PE (IgG1) and appropriate IgG1 isotype controls (BD Biosciences, Erembodegem, Belgium). These combinations were used to identify B-cells (CD20), naïve B-cells (CD20+CD27−), memory B-cells (CD20+CD27+), T independent B-cells (CD20+ IgM+) and T dependent B-cells (CD20+IgM−) [17].

T-cells subsets were stained with mouse anti-human monoclonal antibodies anti-CD45 PeCP, anti-CD3 (IgG1)-PE, anti-CD45RO-FITC (IgG2a), anti-CD27-APC (IgG1) and appropriate IgG1 isotype controls (BD Biosciences, Erembodegem, Belgium). These combinations were used to identify T-cells (CD3), naïve T-cells (CD3+CD45RO−CD27+), early effector/memory T-cells (CD3+CD45RO+CD27+) and late effector/memory T-cells (CD3+CD45RO+CD27−) [18]. For the staining of B-cells, 50 µl of fixed blood was pipetted in two test tubes. Blood in both tubes was washed twice with 2 ml of phosphate buffered saline (PBS) containing 1% bovine serum albumin (BSA) to remove serum. Subsequently, a cocktail of anti-CD20/anti-CD27/anti-IgM was added to one tube and anti-CD20/isotype-control cocktail to the other. After 30 minutes of incubation, red blood cell lysing solution was added for 10 minutes, cells were washed and analysed on the flow cytometer (FACSCalibur, BD Biosciences). For the staining of the T-cells the procedure was the same with exception of the washing step with PBS-BSA which was omitted. The cells subsets were analysed using FlowJo software (Tree Star, US).

Prior to the study, the antibody cocktails were tested using whole blood from 3 normal controls. Blood collected in Cyto-Chex BCT blood collection tubes was compared to fresh blood collected in EDTA tubes. Using the above described antibody cocktails, T- and B- cell subsets could be measured in blood collected in Cyto-Chex BCT blood collection stored for at least 14 days.

### Determination of IgM and IgG antibody titres against red blood cells (iso-agglutinin end-titers)

Screening for irregular anti- erythrocyte antibodies (antibodies causing agglutination but that are not A and B red blood cell carbohydrate antigen specific) was performed with ID-Diacell I–II–III (Bio-Rad, Cressier, Switzerland) using undiluted plasma. For IgG, 25 µl of plasma and 50 µl of ID-Diacell I, ID-Diacell II or ID-Diacell III cell suspension were incubated for 15 minutes at 37°C on Coombs anti-IgG ID-cards (Bio-Rad, Cressier, Switzerland). For IgM, 25 µl of plasma and 50 µl of each cell suspension were incubated for 15 minutes at 20°C on ID-cards NaCl, enzymetest and cold agglutinins (Bio-Rad, Cressier, Switzerland). After incubation, gel cards were centrifuged (ID-centrifuge, Bio-Rad, Cressier, Switzerland) for 10 minutes and the agglutination reaction was scored. Samples positive for irregular anti-erythrocyte antibodies, implying a risk for false positive iso-agglutinin reactions, were excluded from further analysis.

For assessment of antibody titres against A and B red blood cell carbohydrate antigens, plasma samples of patients with blood group O were tested with A1 and B cells (ID-Diacell ABO, Bio-Rad, Cressier, Switzerland), those from blood group A or B were tested with respectively B or A1 cells only, those from blood group AB were not tested. Two-fold serial dilution series of plasma were prepared in phosphate buffered saline (Yvsolab, Turnhout, Belgium). For IgG iso-agglutinin, 25 µl of diluted plasma and 50 µl of ID-Diacell A1 and/or B cell suspension were incubated for 15 minutes at 37°C on Coombs anti-IgG ID-cards (Bio-Rad, Cressier, Switzerland). For IgM iso-agglutinin, 50 µl of diluted plasma and 50 µl of ID-Diacell A1 and/or B cell suspension were incubated for 15 minutes at 20°C on ID-cards NaCl, enzymetest and cold agglutinins (Bio-Rad, Cressier, Switzerland). After incubation, gel cards were centrifuged (ID-centrifuge, Bio-Rad, Cressier, Switzerland) for 10 minutes and the agglutination reaction was scored. The end-titre was the highest plasma dilution still causing an agglutination reaction.

### Measurement of measles antibodies

Quantitative and qualitative determination of specific IgG antibodies to measles virus was performed using Enzygnost anti-measles Virus/IgG ELISA (Siemens, Marburg, Germany), following the manufacturer instructions for the BEP III system (Siemens, Marburg, Germany). Plasma of HAT patients at inclusion and 6 months post-treatment, and corresponding plasma from the respective control were analysed in the same ELISA plate. Based on the reference included in the kit, results were expressed as mIU/ml. Samples with OD<0.1 were negative, samples with OD>0.2 were positive, samples in the grey zone with 0.1<OD<0.2 were retested. A measles antibody level of ≥200 mIU/ml is assumed to provide protection against infection in a healthy population [12], [19].

### Statistical analysis

For analysis, only results for which the corresponding matched sample result at the same time point was available were taken into account. Comparisons of quantitative results between controls and HAT patients and between 0 and 7 months were performed with the Wilcoxon Signed Rank Test (SigmaPlot 11). Comparisons of quantitative results between first and second stage patients were performed with the Mann-Whitney Rank Sum test. Data are presented as medians with interquartile range (IQR). Differences in proportions between controls and HAT patients were assessed with McNemar Chi square (STATA 10.0). A *p*-value of ≤0.05 was considered as significant.

## Results

### Study population

In total, 117 controls and 117 *gambiense* HAT patients were included. Median age was 28 years, 45% of the participants were male. Respectively 9.9% of participants suffered from malaria (13 HAT patients and 9 controls positive/223 thick blood films) and 1 control had HIV. Overall vaccination coverage reported by the study population ranged between 88.4% (183/207) for polio and 100% for BCG, and 80.6% (183/227) of participants had a BCG scar. The general condition for all study participants was judged good to normal. Among the participants, 51.3% had blood group O, 30.2% A, 15.5% B and 3.0% AB. For none of the above parameters, there were significant differences in proportions between HAT patients and controls, except for polio vaccination, reported by 83.3% of controls versus 93.3% of HAT patients (*p* = 0.002).

Among the HAT patients, 97.4% (114/117) were positive in CATT on whole blood (median plasma titre 16, IQR 8–16), 77.4% (48/62) had trypanosomes in the lymph node fluid after successful lymph node puncture, and respectively 43.4% (36/83) and 89.3% (100/112) had trypanosomes in blood detected by the micro-haematocrit centrifugation technique or in the mini-anion exchange centrifugation technique. Cerebrospinal fluid median white blood cell counts were 5/µl (IQR 2–43) and trypanosomes were observed during the cell count in 15.7% (18/115). About half (56/116) of the included HAT patients were in the meningo-encephalitic disease stage (>5 white blood cells/µl or trypanosomes in cerebrospinal fluid).

Respectively 111/117 HAT patients and 105/117 controls were revisited after a median of 211 (IQR 197–241 days, T = 7 months) and 204 days (IQR 178–246) respectively. At revisit, all participants were in good general condition. Although one control had become CATT positive, no trypanosomes were detected in any of the study participants.

### Memory B-cells during and after HAT

**Figure 1:**
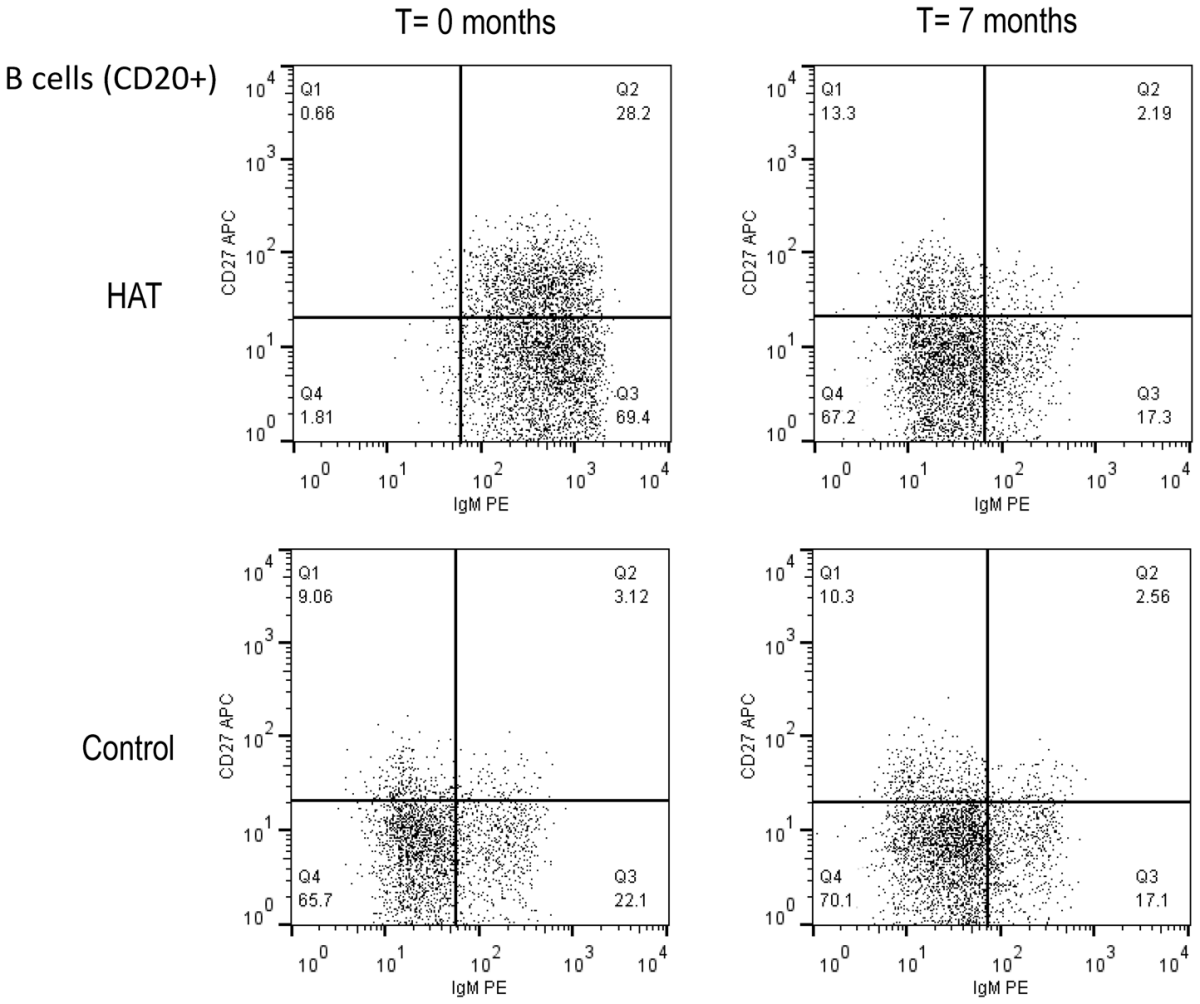
Flow cytometry dot plot of the CD20+ B-cell population in HAT and in a control. B-cell subsets were based on the CD27 and IgM cell surface markers. A HAT patient before and after treatment (T = 0 and 7 months), and a healthy control subject at the same time points are shown. Cut-offs for considering a cell surface marker positive or negative were based on isotype controls and are shown as solid lines, and subdivide the graph into 4 quadrants (Q1–Q4). B-cell subsets in each quadrant are expressed as percentages of CD20+ B-cells.

An overview of the B-cell phenotyping results in HAT patients and controls is presented in Table 1, and an example of a dot plot of CD27 and IgM expression on B-cell subsets (CD20+), in a HAT patient and a control is shown in Figure 1. The percentage of CD20+ B-cells in HAT patients was significantly higher than in controls (median 1.5 times higher, *p*<0.001). Although the percentage of CD20+ cells had decreased 6 months after treatment of HAT, it still remained significantly higher than in controls (*p*<0.001). Within the CD20+ subset, the percentages of CD27+ memory B-cells and IgM+ B-cells were significantly higher in HAT than in controls (increases of the median of respectively 2.3 and 3.6 times, *p*<0.001). After HAT treatment the percentage of CD27+ cells within the B-cell (CD20+) subset still remained significantly higher than in controls (*p* = 0.001), while no significant difference could be observed anymore for the percentage of IgM+ B-cells (*p* = 0.7). The most striking change within the B-cells subset was the more than 6-fold increase of the percentage CD27+IgM+ cells (Q2 in Figure 1) in HAT patients compared to controls (*p*<0.001). After treatment, this subset returned to normal percentages. HAT was associated with only minor differences in the CD27+IgM− subset (Q1 in Figure 1, *p* = 0.004) of B cells. The relative decrease of naive (CD27−) B-cells in HAT was mainly due to a decrease of CD27−IgM− cells (Q4 in Figure 1, *p*<0.001) while the percentage of CD27−IgM+ cells within the B-cell subset had increased (Q3 in Figure 1, *p*<0.001). For none of the B-cell phenotypes studied, significant differences between stage 1 and stage 2 HAT patients were observed (0.2<*p*<0.9).

**Table 1:** Peripheral blood B-cell subsets in HAT and in controls.

% B-cell subset		Median (IQR), T = 0 months, n = 84	Median (IQR), T = 7 months, n = 70	*p*
**B-cells (CD20+)**	Control	11.4 (8.0–14.3)	10.5 (7.7–13.2)	0.9
	HAT	17.0 (12.8–22.4)	14.0 (10.6–19.0)	<0.001
	*P*	<0.001	<0.001	
**Naive B-cells (CD27−)**	Control	86.9 (81.5–90.8)	81.2 (73.3–86.5)	0.001
	HAT	69.6 (58.7–79.3)	74.5 (62.7–83.4)	0.4
	*P*	<0.001	0.003	
**Memory B-cells (CD27+)**	Control	13.0 (9.2–17.8)	18.3 (12.2–24.3)	0.003
	HAT	30.1 (20.8–40.9)	24.6 (16.2–36.6)	0.5
	*P*	<0.001	0.001	
**T dependent B-cells (IgM−)**	Control	86.0 (65.0–94.6)	84.3 (74.4–91.8)	0.03
	HAT	50.3 (22.1–69.3)	83.8 (75.6–94.9)	<0.001
	*P*	<0.001	0.9	
**T independent B-cells (IgM+)**	Control	13.7 (4.5–34.8)	14.2 (5.2–23.8)	0.009
	HAT	49.2 (30.7–77.3)	15.8 (3.6–23.9)	<0.001
	*P*	<0.001	0.7	
**T dependent memory B-cells (CD27+IgM−), Q1**	Control	10.9 (7.2–14.4)	15.8 (11.7–20.5)	<0.001
	HAT	12.7 (7.9–22.1)	21.4 (14.4–32.0)	<0.001
	*P*	0.004	0.002	
**T independent memory B-cells (CD27+IgM+), Q2**	Control	2.0 (0.7–4.5)	2.4 (1.1–3.9)	0.5
	HAT	13.0 (6.3–21.9)	2.5 (0.8–5.9)	<0.001
	*P*	<0.001	0.1	
**T independent naive B-cells (CD27−IgM+), Q3**	Control	11.9 (3.0–24.4)	9.11 (2.8–16.1)	0.004
	HAT	35.4 (14.2–47.7)	9.8 (1.6–15.4)	<0.001
	*P*	<0.001	0.5	
**T-dependent naive B-cells (CD27−IgM−), Q4**	Control	72.9 (55.7–85.5)	67.9 (62.8–76.6)	0.8
	HAT	31.9 (17.4–49.1)	65.7 (52.7–72.7)	<0.001
	*P*	<0.001	0.005	

Blood was taken from HAT patients and from non-HAT controls before treatment (T = 0 months) and after treatment or at the corresponding time point (T = 7 months). All comparisons were performed with the Wilcoxon Signed Rank Test. B-cells are expressed as percentage of all lymphocytes and all the B-cell subsets are expressed as percentages of B-cells. Q1–4 refers to the corresponding quarters in Figure 1. Individual data are shown in Fig. S1, and can be obtained from the authors upon request.

### Memory T-cells during and after HAT

**Figure 2:**
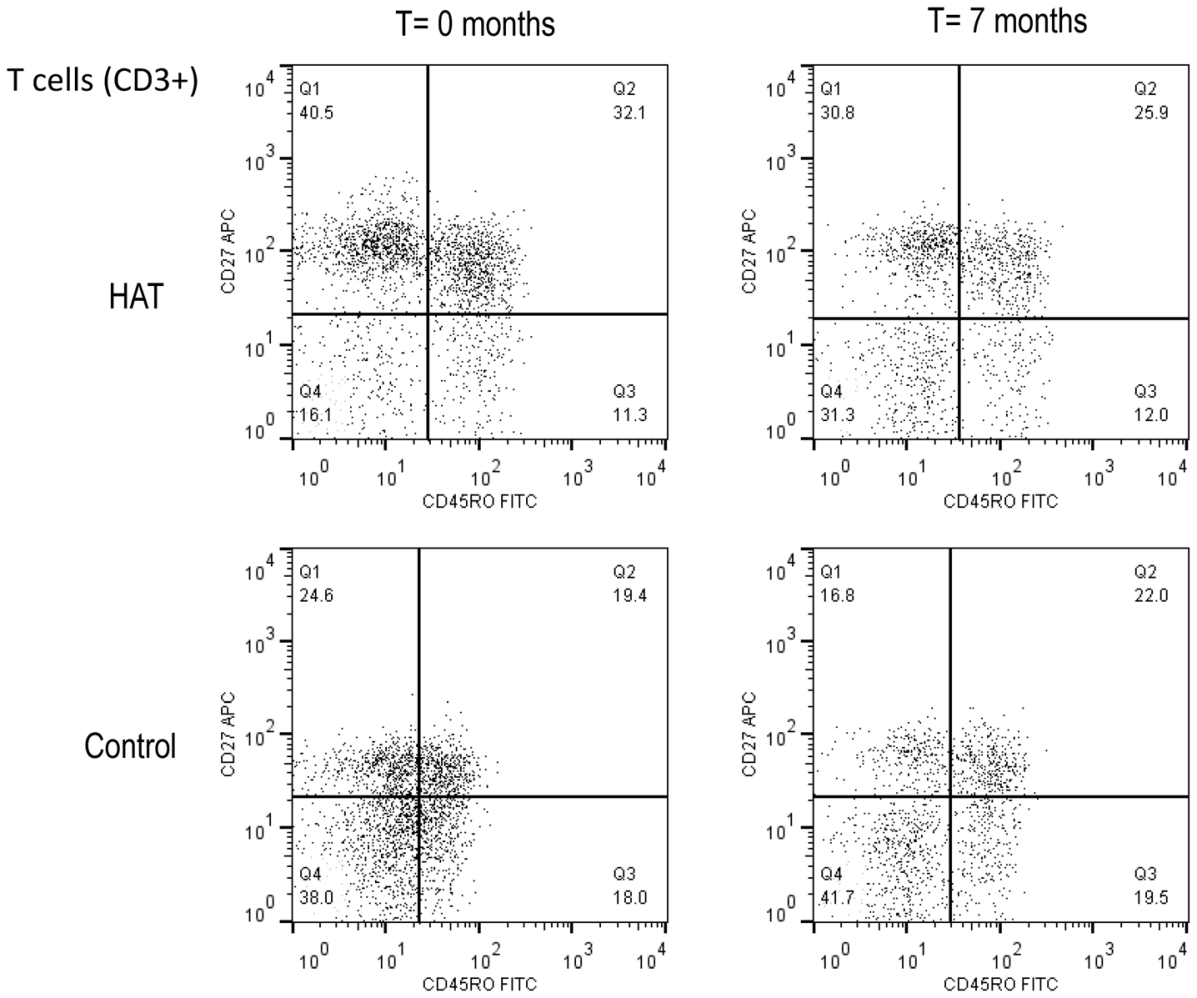
Flow cytometry dot plot of the CD3+ T-cell population in HAT and in a control. T-cell subsets were based on the CD27 and CD45RO cell surface markers. A HAT patient before and after treatment (T = 0 and 7 months), and a control at the same time points are shown. Cut-offs for considering a cell surface marker positive or negative are shown as solid lines, and subdivide the graph into 4 quadrants (Q1–Q4). T-cell subsets in each quadrant are expressed as percentages of CD3+ T cells.

A summary of the T-cell phenotypes is presented in Table 2, and an example of a dot plot of the CD27 and CD45RO expression on T-cell subsets (CD3+), in a HAT patient and a healthy control subject is shown in Figure 2.

**Table 2:** Peripheral blood T-cell subsets in HAT and in controls.

% T-cell subset		Median (IQR), T = 0 months, n = 84	Median (IQR), T = 7 months, n = 70	*p*
T-cells (CD3+)	Control	73.2 (66.2–76.7)	71.2 (68.0–75.7)	0.02
	HAT	66.7 (61.3–73.1)	70.4 (64.4–75.9)	0.001
	*P*	<0.001	0.3	
Memory T-cells (CD45RO+)	Control	39.7 (31.1–48.5)	42.2 (33.5–50.5)	0.9
	HAT	44.6 (38.9–51.2)	43.4 (37.0–49.7)	<0.001
	*P*	0.002	0.2	
Naive T-cells (CD45RO−CD27+), Q1	Control	28.0 (20.7–39.6)	25.5 (14.6–37.1)	0.5
	HAT	28.1 (19.3–39.7)	27.0 (15.4–34.8)	0.3
	*P*	0.8	0.8	
Early effector/memory T-cells (CD45RO+CD27+), Q2	Control	16.7 (13.0–20.4)	13.4 (8.4–19.1)	0.09
	HAT	19.4 (14.9–24.7)	15.0 (10.2–18.9)	<0.001
	*P*	0.003	0.5	
Late effector/memory T-cells (CD45RO+CD27−), Q3	Control	23.0 (17.7–27.7)	25.4 (18.2–34.9)	0.05
	HAT	19.5 (16.2–26.1)	27.3 (21.1–34.4)	<0.001
	*P*	0.09	0.1	
Effector T-cells (CD45RO−CD27−), Q4	Control	23.3 (17.8–32.5)	24.1 (17.2–33.2)	0.7
	HAT	18.1 (14.4–23.9)	23.6 (19.2–32.1)	<0.001
	*P*	<0.001	0.8	

Blood was taken from HAT patients and non-HAT controls at inclusion (T = 0 months) and after treatment or at the same time point (T = 7 months). All comparisons were performed with the Wilcoxon Signed Rank Test. T-cells expressed as percentage of all lymphocytes. All the T-cell subsets are expressed as percentages of T-cells. Q1–4 refers to the corresponding quarters in Figure 2. Individual data are shown in Fig. S2, and can be obtained from the authors upon request.

The percentage of CD3+ T-cells was significantly lower in HAT than in controls, and returned to normal 6 months after treatment. Within the T-cells subset, memory T-cells were significantly increased (CD45RO+, *p* = 0.002), which was due to a relative increase in early effector/memory (CD45RO+CD27+) T-cells in HAT (1.2 fold increase of the median, *p* = 0.003, Figure 2 Q2). After treatment, the observed differences in memory T-cell subsets between HAT and controls disappeared. No difference was observed in percentage of naïve (CD45RO− CD27+) T-cells between HAT patients and controls (p = 0.8) while the percentage of late effector (CD45RO−CD27−) T-cells was significantly lower in HAT than in controls (*p*<0.001, Figure 2 Q4), but normalized after treatment.

No differences were observed in function of the disease stage for any of the measured T-cell subsets (p>0.08).

### IgG and IgM iso-agglutinin end-titres

Screening for irregular anti-erythrocyte IgG with ID-Diacell I–II–III cells revealed respectively 9/116 and 12/104 reactive controls at T = 0 months and T = 7 months (9 at both time points), and 7/116 and 5/109 reactive HAT patients (4 at both time points). At T = 0 months or T = 7 months, respectively, 7/116 and 4/104 controls (4 at both time points), and 6/116 and 5/109 HAT patients (4 at both time points) reacted for irregular anti-erythrocyte IgM. At inclusion, there was no difference in anti-A or anti-B IgG end titers between controls and HAT patients (table 3). For IgM, at time of inclusion median anti-A1 and anti-B iso-agglutinin end-titres were significantly lower in HAT patients than in controls (*p*<0.004). After treatment, at T = 7 months, the anti-A1 IgM iso-agglutinin end-titre had increased significantly in HAT patients (*p*<0.01), but remained lower for anti-B IgM.

**Table 3:** Iso-agglutinin end-titres in HAT and in controls.

Parameter		Median (IQR), T = 0 months	Median (IQR), T = 7 months	*P*
**Anti-A1 IgG end-titre**	Control	128 (64–256)	128 (64–256)	a
	HAT	64 (16–256)	128 (64–256)	a
	*P*	0.1 (n = 47)	0.7 (n = 31)	
**Anti-B IgG end-titre**	Control	64 (32–256)	64 (32–256)	0.2
	HAT	64 (16–128)	64 (32–128)	0.9
	*P*	0.6 (n = 64)	0.7 (n = 58)	
**Anti-A1 IgM end-titre**	Control	128 (64–256)	128 (64–256)	0.5
	HAT	64 (16–128)	128 (32–256)	0.01
	*P*	0.004 (n = 46)	0.5 (n = 33)	
**Anti-B IgM end-titre**	Control	64 (32–256)	64 (32–256)	1
	HAT	32 (16–64)	64 (16–128)	0.08
	*P*	0.002 (n = 67)	<0.001 (n = 63)	

Blood was taken from HAT patients before and after treatment (T = 0 and T = 7 months) and from non-HAT controls at the same time points. n: number of matched pairs used for comparison.

a Not calculated due to data loss. Full data can be obtained from the authors upon request.

There was no difference in iso-agglutinin end-titres between stage 1 and stage 2 HAT patients (*p* values>0.1), except for anti-B IgM which was one titer lower in stage 2 (*p* = 0.05).

### Antibody levels against measles

**Figure 3:**
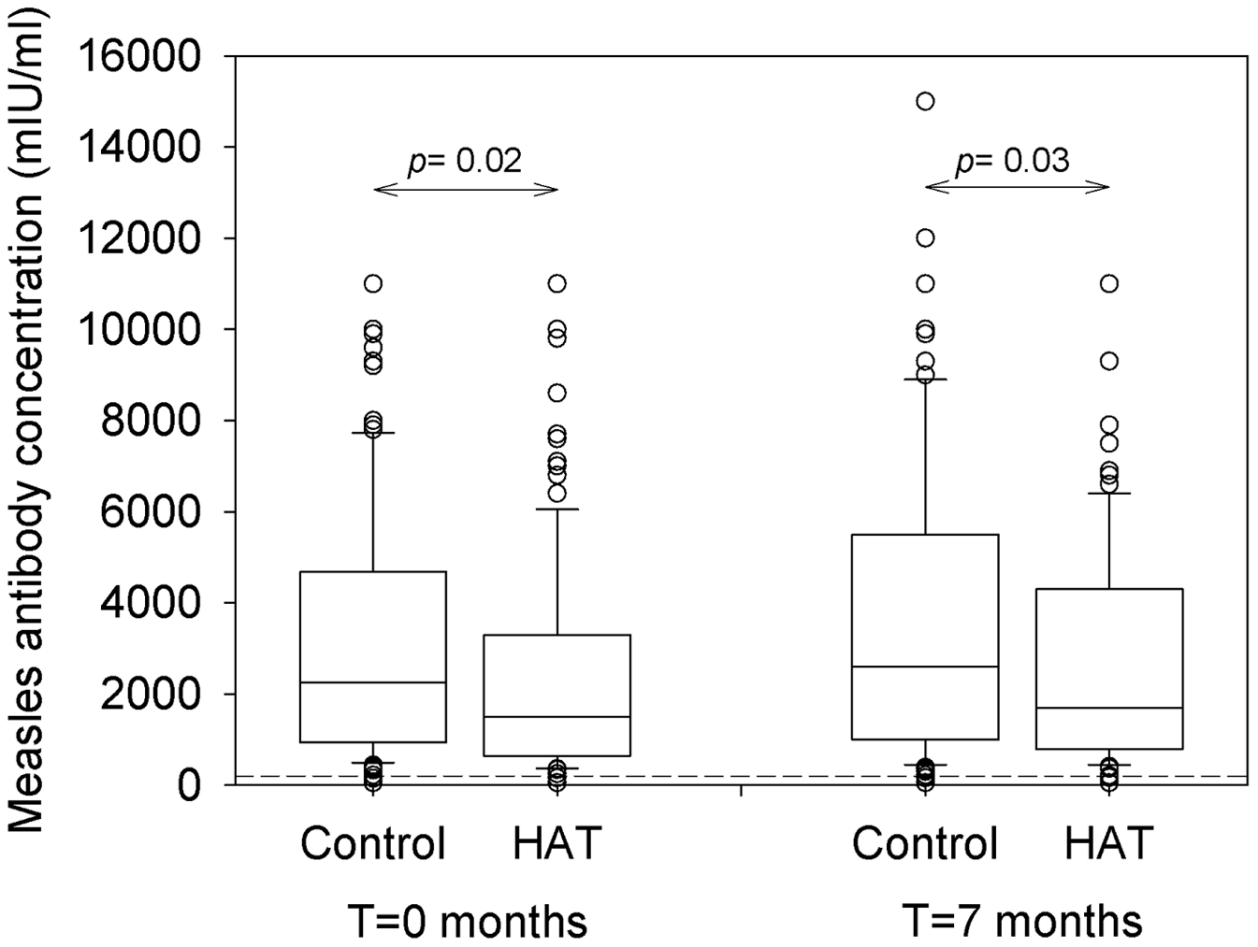
Box plot of measles antibody concentrations in HAT and controls. Blood was taken from HAT patients and non-HAT controls before treatment (T = 0 months, n = 116), and after treatment or at the same time point (T = 7 months, n = 99). The cut-off value for protective immunity of ≥200 mIU/ml is indicated by the dashed line. Full data can be obtained from the authors upon request.

Measles antibody concentrations in HAT patients at inclusion and after treatment and in controls at corresponding time points are summarized in Figure 3. At inclusion, the median antibody concentration in HAT patients (1500 mIU/ml, IQR 643–3300) was significantly lower than in controls (2250 mIU/ml, IQR 940–4675). Seven months later, the antibody concentration in the treated HAT patients (1700 mIU/ml, IQR 790–4300) remained significantly lower than in controls (2600 mIU/ml, IQR 1000–5500) although in both groups, the antibody level had increased significantly compared to inclusion (*p*<0.001 and *p* = 0.006 respectively). There was no difference in measles antibody concentration between stage 1 and stage 2 HAT (*p* = 0.7).

A measles antibody level superior to the cut-off assumed to provide protection against infection was present in 94.8% (110/116) of HAT patients and in 98.3% (114/116) of controls, at inclusion and 7 months later. There was no difference in proportions of HAT patients and controls exceeding this cut-off (*p* = 0.3).

No relationship between high measles antibody levels and self-reported vaccination against measles, polio, diphtheria-tetanus-pertussis, BCG or presence of a BCG scar could be observed (*p* = 0.6–1).

## Discussion

Our results suggest that the issue of B-cell dysfunction that troubles mouse models for trypanosomiasis, might not be that severe in human African trypanosomiasis patients infected with *T.b. gambiense*. In *gambiense* HAT patients compared to controls, significantly higher percentages of memory B- and memory T-cells were present in peripheral blood. After treatment, the percentage of memory T-cells normalized and the percentage of memory B-cells did not yet normalize. Iso-agglutinin IgM end-titres were slightly lower in *gambiense* HAT, and normalized only partially after treatment. Although anti-measles antibody levels were, and remained, lower in *gambiense* HAT patients than in controls, no significant difference could be observed in the number of individuals with levels above the international cut-off for protection.

Memory cell populations in experimental *T.b. brucei* infection have exclusively been studied in bone marrow and spleen [5]. For *T. vivax* experimental mice infections, peripheral blood data are available as well [20]. In the human host, only peripheral blood is readily accessible. Until now, data on peripheral blood lymphocyte subsets in HAT are rare, due to the important logistic challenges related to conducting research in settings like DR Congo. The observed relative B-cell increase is consistent with previous findings [10] and in line with polyclonal B-cell activation and proliferation of cells of the B lymphoid series previously described for HAT [21]. The upregulation of Fas (CD95) expression in *gambiense* HAT, measured by Boda *et al.*, led these authors to suggest a poor conversion of B-cells into memory B-cells [10]. In our study, we observed a relative increase of CD27+IgM+ B-cells which are defined as T independent memory B-cells [17] in HAT. In *T. vivax* experimental mice infections, the fall in number of B-cells in the lymphoid organs is similar to experimental *T.b. brucei* infections. In peripheral blood it is accompanied by an increase in the number of transitional IgM+IgD− B-cells and switched IgM−IgD− plasma/memory cells and by a decrease in naive B-lymphocytes [20]. Although the marker combinations used to identify B-cells in these experimental *T. vivax* studies were different from ours, the results for peripheral blood are similar, if we assume that the memory B-cells defined by CD27+ in HAT, are similar to the IgD− population in *T. vivax* infected mice [17].

We confirm the moderate relative T-cell decrease in *gambiense* HAT observed previously by Boda *et al.*, associated to the relative expansion of B-cells. The present observation of a relative increase in early memory T-cells (CD45RO+CD27+) seems to corroborate earlier findings of larger numbers of CD4+CD45RA−CD62L+ cells in HAT with CD8+CD45RA−CD62L+ cells remaining constant [10]. As previously suggested [10], there were no differences in lymphocyte subsets according to the disease stage.

HIV also causes a significant increase in the memory (CD45RA−CD45RO+) phenotype CD8 subset [22] and reduces the CD27+ memory B-cell population [23]. The HIV prevalence in our study population was low and is not expected to affect the overall results. Malaria, which is associated with B- and T-cell exhaustion and an increase in an atypical CD19+CD27−CD21−CD10− memory B-cell population [24], is not expected to account for differences between the control and HAT population since the frequency of occurrence of trophozoites in blood was similar in both groups. However, malaria, or other infections, might account for variation in some cell phenotypes in time, as was observed in the control group. This underlines the importance to sample controls at similar time points as HAT patients and to perform a matched statistical analysis, to maximally eliminate external variation.

The loss of the host's capacity to recall vaccine-induced memory responses, as has been described for laboratory animals [6], [7], can in humans, for ethical reasons, not be tested by challenge with a pathogen. Therefore, the Multitest cell-mediated immunity (Pasteur-Mérieux, Lyon, France), an intradermal skin test to measure delayed hypersensitivity as a marker for T-lymphocyte response, was considered but this test was no longer available anymore at time of the project. Neither was it feasible to set-up of facilities for cell culture or ELISPOT. We therefore had to rely on surrogate markers and opted for the quantification of iso-agglutinins and measles antibodies.

Natural IgM antibodies against A and B carbohydrate antigens are T-cell independent, while a T-cell dependent antibody response results in higher affinity IgG1 and IgG3 antibodies [11]. In the presence of an intact immune system, iso-agglutinins to the missing A or B red blood cell carbohydrate antigens are always found, even if there has been no exposure to red blood cells carrying these antigens. These antibodies were therefore used to asses T-cell independent and T-cell dependent humoral immunity. The lower IgM iso-agglutinin titers observed in HAT patients, might indeed point to a moderate effect of *gambiense* HAT on the T-independent antibody response [6], but seems reversible upon treatment.

Measles were selected for antibody quantification for the following reasons. A high proportion of the population is expected to have antibodies against measles since the measles vaccine is part of the standard vaccination programs [12]. Half-life of measles IgG antibodies has been estimated at around 3000 years so they should be measurable in all subjects that have been infected or were successfully immunized [25]. Moreover, in healthy individuals, the absolute level of antibodies needed to fully protect against infection is known, as well as the concentration below which no protection is obtained anymore [26], [27]. Vaccine coverage in the last 33 years in DR Congo has been estimated by WHO-UNICEF as 21–95% for BCG and 17–90% for the measles vaccine respectively [28]. Presence of a BCG scar in 80.6% of study participants indicated rather high vaccine coverage, and measles antibody levels above the cut-off were present in 96.6%. Measles antibody levels were comparable to levels observed in pregnant women in Belgium and in a Swedish volunteer group, in which concentrations were measured with the same ELISA kit [29], [30], or in an adult population in Addis-Abeba [31]. The increase in measles antibody concentrations in HAT patients and controls 7 months after inclusion, is unlikely due to a technical bias since samples taken at both time points were analyzed in the same ELISA plate. The rise might have been caused by a natural exposure to measles, as measles outbreaks regularly occur in DR Congo, also during the study period [32]. Although existing data are contradictory, presence of antibodies does not necessarily reflect presence of antibody secreting memory B-cells, as continuous antibody secretion might be due to long-lived plasma cells rather than on-going activation of memory B-cells [30]. Measles vaccine induces both humoral and cellular immune responses comparable to those following natural infection [33]. We withheld from revaccination, since live virus measles vaccination is not recommended for immune-suppressed patients [27], a condition to be expected during HAT, and since revaccination was judged not to be in the patient's best interest, even after treatment for HAT.

Overall, our results do not exclude an impairment of humoral and cellular immunity during *gambiense* HAT. Indeed, when given during *gambiense* HAT infection, a reduced response to typhoid vaccine has been observed, as well as diminished reactions to skin test antigens [34]. Similar observations have been made in domestic animals, where the antibody response to and/or efficacy of vaccination against *e.g.* contagious bovine pleuropneumonia [35], foot and mouth disease [36], swine fever [37], antrax spore [38] and *Brucella abortus* [39] were affected when given during infection with various animal trypanosomes. Moreover, in the immunized *T.b. brucei* - *Trichinella spiralis* co-infection experimental model, the anti-*Trichinella* IgG1 response was not affected [7] although protection was partially lost. Due to polyclonal B-cell activation, characteristic for trypanosomiasis infection, specific functional antibodies may be replaced by non-protective, low affinity, cross-reactive antibodies [40]. Although for measles, antibody concentrations remained above the cut-off, we cannot exclude that they have become unfunctional in HAT and their protective capacity may have been lost. The lack of a functionality test is an important difference with previously published experimental mice studies [6], [7] and represents the main limitation of the actual study. It might therefore be worth to further assess the protective capacity of the measles antibodies against infection, *e.g.* using a functional assay such as the plaque reduction neutralization test [41]. However, none of the study participants mentioned a measles episode while being questioned for their vaccination history, although as mentioned above, natural exposure might have occurred. Of interest, the agglutinating capacity of the iso-agglutinin antibodies was only moderately affected in our assay.

As discussed above, other limitations inherent to our study are mainly related to research in humans instead of in laboratory animals, and to studying a disease that typically occurs in rural Africa, far from high-tech environments. In this context, blood specimens were collected on a blood stabilizer, intended to preserve peripheral blood samples' qualitative and quantitative leukocyte subset characteristics and allowing collection and storage of blood specimens for immunophenotyping by flow cytometry. Even using this stabiliser, preliminary experiments demonstrated that some lymphocyte subset cell markers (*e.g.* the cell surface marker CCR7, which we had originally selected to be used in combination with CD27 to better identify T memory subsets [18] were not optimally preserved, thus antibody cocktails had to be adapted accordingly. As we did not perform absolute counting of lymphocyte numbers, the observed changes in lymphocyte sub-populations are relative. For the iso-agglutinins, the participants blood group has to be taken into account, but the blood group was not used as a matching criterion at time of collection. In the settings we were working, in practice, it would have been difficult to identify a matched control for the patients with rarer B and AB blood groups. Data for HAT patients and controls that had different blood groups and were not tested against the same red blood cell carbohydrate antigen, were therefore lost. However, similarly lower IgM end-titers in HAT patients were also observed when statistical analysis was performed without matching the results for HAT patients and their corresponding controls (data not shown).

Overall, our results in *gambiense* HAT patients do not suggest trypanosomiasis associated massive memory cell destruction, or loss of antibody levels, although the antibody's protective capacity remains to be confirmed. So far there have never been epidemiological signals/reports that HAT patients, before or after treatment, were at increased risk of having vaccine-preventable diseases (measles or others) compared to the rest of the population. One should however acknowledge that epidemiological surveillance is generally weak in rural Africa and that such occurrences might have been missed. If some degree of immunity loss may exist in HAT patients infected with *T.b gambiense*, it does not seem of clinical relevance. At least for measles, our data indicate that antibody levels remain intact. Some open questions remain. Functionality of measles antibodies should be confirmed to completely ensure that revaccination after gambiense HAT, would not be necessary. It could also be interesting to assess activity of other vaccine-induced antibodies, as the decay of measles antibody concentrations is extremely slow and since we cannot exclude that other vaccines might depend more on memory cell dependent antibody production. Differences in immune-suppression and B-cell apoptosis observed between *gambiense* HAT and experimental infections may be linked to the differences in parasitemia between *T.b. gambiense* HAT and experimental infections [5], [34]. As previously suggested [5], [34], it might therefore be worth to perform similar investigations in acute *T.b. rhodesiense* HAT, which is characterized by higher parasitemia, and for which no data on peripheral blood memory T- and B-cells or on acquired immunity are available.

## Supporting Information

Figure S1 Peripheral blood B-cell subsets in HAT and in controls. Blood was taken from HAT patients and from non-HAT controls before treatment (T = 0 months, n = 84) and after treatment or at the corresponding time point (T = 7 months, n- = 70). B-cells are expressed as percentage of all lymphocytes and all the B-cell subsets are expressed as percentages of B-cells.
(TIF)

Figure S2 Peripheral blood T-cell subsets in HAT and in controls. Blood was taken from HAT patients and non-HAT controls before treatment (T = 0 months, n = 84) and after treatment or at the same time point (T = 7 months, n = 70). T-cells are expressed as percentage of all lymphocytes. All the T cell subsets are expressed as percentages of T cells.
(TIFF)

Figure S3 Measles antibody concentrations in HAT and controls. Blood was taken from HAT patients and non-HAT controls before treatment (T = 0 months, n = 116), and after treatment or at the same time point (T = 7 months, n = 99).
(TIF)
